# The utilisation of Complementary and Alternative Medicine (CAM) among ethnic minorities in South Korea

**DOI:** 10.1186/1472-6882-14-103

**Published:** 2014-03-19

**Authors:** Jung Hye Hwang, Dong Woon Han, Eun Kyung Yoo, Woon-Yong Kim

**Affiliations:** 1 Department of Obstetrics and Gynecology, Hanyang University College of Medicine, 222 Wangsimni-ro, Seongdong-gu 133-791, Seoul, Korea; 2 Department of Global Health and Development, Graduate School, Hanyang University, 222 Wangsimni-ro, Seongdong-gu 133-791, Seoul, Korea; 3 Department of Preventive Medicine, Hanyang University College of Medicine, 222 Wangsimni-ro, Seongdong-gu 133-791, Seoul, Korea; 4 Institute of Health Services Management, Hanyang University, 222 Wangsimni-ro, Seongdong-gu 133-791, Seoul, Korea

## Abstract

**Background:**

Race has been reported to affect the use of complementary and alternative medicine (CAM), but there is very little research on the use of CAM by ethnicity in Korea. This study explores the prevalence of CAM use among ethnic minorities in South Korea.

**Methods:**

The design is a descriptive and cross-sectional study. A convenience sample of ethnic minorities was recruited from two public healthcare centres in Gyeonggi province. The survey instrument included 37 questions regarding CAM use, factors influencing use of CAM, self-health management, and the socio-demographic profile of study participants.

**Results:**

Sixty-two percent of study participants reported the use of CAM. Multivitamins (53.3%), acupuncture (48.9%), and traditional Korean herbal medicine (38.9%) were popular CAM modalities in our sample. Other notable CAM modalities included herbal plants, therapeutic massage, and moxibustion therapy. The majority of CAM users (52.2%) received CAM services to treat diseases or as a secondary treatment while receiving conventional care. Having positive perceptions toward the effectiveness of CAM was a major determining factor in CAM use.

**Conclusions:**

Physicians need to be aware of the fact that many ethnic minorities use CAM therapies. Many CAM users reported that they want doctors to know about their CAM use and have a basic understanding of traditional medicine in their home country. Overcoming language and cultural barriers will help reduce unwanted medical complications. High prevalence of CAM use among ethnic minorities in our study warrants further studies using larger sample population.

## Background

The recent surge in immigration has set the foundation for South Korea to become a multi-cultural society. As other nations have experienced, increasing numbers of ethnic minority communities have adverse effects in many facets of society. One area that is heavily affected is the healthcare sector. Apart from heavily discussed issues such as disparity in healthcare access, past U.S. studies have documented the link between the underutilisation of complementary and alternative medicine (CAM) and ethnic minority status [1-7]. In studies in the United States, ethnic minorities were less likely to use CAM modalities, and the overall prevalence of CAM use was on average 10-20 percent lower than in non-Hispanic Whites [4,6,8].

While earlier studies shed a light on the potential disparity in access to CAM services among ethnic minorities, the findings from these studies are not fully convincing because they use nationally representative data that do not oversample ethnic minorities, possibly leading to overgeneralised results [5,8,9]. Using a smaller national data set that oversamples minorities, Mackenzie et al. has shown that CAM use in ethnic minority groups was not different compared to non-Hispanic Whites [8]. This was possibly because oversampled minorities in the survey allowed a better representation of CAM users within that group. Findings from earlier studies of CAM use among ethnic minorities were also limited in that they used broadly defined categories of race/ethnicity that hardly reflect diversity of this group [5,9]. A recent study used ethnic-specific measures of CAM use and refined categories of race/ethnicity in an effort to better represent the diversity within the ethnic minority population [9]. Hsiao et al. [9] found that CAM use varied within ethnic minority groups, and each ethnic group had certain types of CAM modalities that were favoured over others.

While most of these studies were conducted in the U.S. where ethnic diversity is already well established and where there is a rich history of immigration, we do not know much about what effects ethnic minority status might have on the utilisation of CAM and the types of CAM modalities used in countries like South Korea where diversity is a relatively recent phenomenon. When compared to other multi-cultural societies, the composition of Korean ethnic minority communities is very different from what we are familiar with from studies conducted in the U.S. and European countries. With many immigrants coming from China and less-developed surrounding nations, it is highly likely that the pattern of healthcare services and CAM use will show a very different trend. Two recent studies in neighbouring Asian countries, Malaysia and Singapore, showed that the prevalence of CAM use was generally very high among Asian ethnic minorities [10,11]. Siti et al. [11] found that over 90% of surveyed ethnic groups in Malaysia reported use of biologically based therapies and that preferred choice of CAM also varied by ethnicity [11]. In Singapore, overall prevalence of CAM use was 76% and 69% for smaller ethnic groups, namely Malays and Indians [10]. Almost all (99%) of surveyed Chinese in Singapore reported using traditional Chinese Medicine [10].

Although foreign-born ethnic minorities account for a very small percentage of the South Korean population, there has been a dramatic increase in the last decade [12-15]. As of 2011, close to a million foreigners from 220 countries worldwide were documented in South Korea, up four-fold from 2000 [12]. Foreign-born minorities in South Korea include but are not limited to the following: Chinese immigrants, both less-skilled and skilled migrant workers from developing countries in Southeast Asia and Central Asia, foreign brides from Southeast Asia, and English instructors from English-speaking countries [12,13,16-18].

Unfortunately, not much information is available on CAM use among ethnic minorities in South Korea. Because a large proportion of ethnic minorities migrating to Korea are not well-educated and are from Asian countries that are still undergoing economic development, they might use CAM modalities in place of or in addition to conventional healthcare services, possibly due to cost and communication barriers in accessing medical care. In an ethnically homogeneous society like South Korea, ethnic minorities’ access to healthcare might be further complicated by differences in health beliefs and varying attitudes towards the health care system [7,19].The lack of availability of such information is concerning, as it may widen the knowledge gap and healthcare disparity of this population. The effectiveness of many CAM modalities is not yet clinically proven. For herb-based naturopathic CAM modalities, adverse reactions that may occur when used with conventional drugs are understudied. Thus, it might also present new dilemmas for Korean clinicians and policy makers as ethnic minorities are less likely to disclose their CAM use with physicians [20].

As the first study to focus on CAM use among ethnic minority groups in South Korea, our study explored the prevalence of CAM use and factors influencing the decision to use CAM modalities. We also paid special attention to the types of CAM modalities used, self-perception toward CAM use, and reasons for using and supplementing CAM with conventional medicine. Although it is not covered by the Korean national health insurance, Korea spends a large sum of medical expenditures on research, development, and traditional Korean medicine (TKM). TKM is a formally accepted CAM in South Korea, and its roots go back decades in Korean history. Methods are inclusive of several CAM modalities, such as acupuncture, moxibustion therapy, and herbal remedies. Thus, we also explore whether TKM is being used by surveyed ethnic minorities.

## Methods

We surveyed ethnic minorities who visited or were admitted to two large public healthcare centres in Gyeonggi province. Although we are aware of the potential bias that might occur in conducting health surveys at healthcare facilities, there is no sampling frame of ethnic minorities in Korea. Public healthcare centres in South Korea offer free non-disclosed consultations and basic medical services to foreigners. The centres also offer vaccination services to foreigners from countries that do not require Japanese encephalitis vaccine and to those who are not up-to-date on hepatitis A and B, diphtheria, tetanus, pertussis (DTP), and typhoid vaccinations. Thus, we have a higher chance of locating potential participants who may have been excluded in a door-to-door survey.

Potential subjects were informed that the purpose of the study and participation was voluntary. Then informed consent to participate was obtained from each of the participants in our study. The survey was distributed to the participants during the month of November 2011. The survey instrument included 37 questions including, but not limited to, questions on self-health management, CAM utilisation, CAM modalities, reasons for choosing or not choosing to use CAM, and socio-demographic profile. In the case where participants had difficulty with Korean language or the survey in general, professional interpreters and social workers from public healthcare centres were assigned on-site to assist participants. The survey was distributed to a total of 360 ethnic minorities, and 292 (Response rate = 81.1%) participated in the study and were included in the final sample for analysis. To ensure the accuracy of data processing, a manual double entry system was applied to input the data. In general, data quality was excellent with only few cases that had too many missing values and omitted sections in the survey. Those cases were excluded from the final analytical sample. Our sample includes participants from countries where traditional medicine is publicly supported and widely recognised as a part of formal healthcare systems, namely, China, Japan, Vietnam, Sri Lanka, and Mongolia. We identified whether CAM receives public support using the following criteria: countries that use public funds in support of schools of traditional medicine, countries that actively participate in CAM research and development, and countries where a large sum of medical expenditure is used on consumption of CAM modalities. We expected that people coming from those countries would have different perceptions toward CAM use. To compare the socio-demographic characteristics of the respondents, and to compare the prevalence of use of each CAM modality and participants’ CAM-related perceptions toward health beliefs and healthcare services, Pearson’s χ^2^-test was used and where necessary the two-tailed Fisher’s exact test was used. All *P value* tests were two-sided. All analysing the data collected from the study were computed using Statistical Package for Social Sciences (SPSS) v. 18.0. Ethical approval was obtained from the Institutional Review Board on Human Subjects Research and Ethics Committees, Hanyang University Guri Hospital (2012-018).

## Results

Table 1 presents the socio-demographic profile of study participants by CAM use status. Overall, 62% (n = 181) of the participants reported using one or more CAM modality. While the largest number of participants was from China, Vietnam, and Philippines, other participants came from 12 other countries, including Cambodia, Pakistan, Japan, and Russia. When examined separately by ethnicity, the prevalence of CAM use varied from 42% to 100%. Among CAM users in our sample were mostly females (74.6%), were under 40 years of age (74%), had less than or equal to a high school education (62.4%), make less than 2 million KRW per month (72.9%), were relatively recent immigrants who stayed for less than 10 years (80.1%), regularly received healthcare services (86.7%), and had insurance (70.7%). In terms of health management characteristics, 79% of CAM users did not exercise regularly and generally had a negative self-perceived health status. The results of univariate logistic regression analysis showed that gender, age, employment status, recent immigration, and receiving regular healthcare were potential predictors of CAM use.

**Table 1:** Socio demographic, healthcare utilisation, and self-health management characteristics of respondents

**Variables**	**Total number of respondents, N (%)**	**CAM utilization status**		***P-value***^***a***^
		**Non-CAM user n (%)**	**CAM user n (%)**	
	**292 (100.0)**	**111 (100.0)**	**181 (100.0)**	
**Socio-demographic characteristics**				
Gender				0.042
Male	63 (21.6)	46 (15.3)	46 (25.4)	
Female	229 (78.4)	94 (84.7)	135 (74.6)	
Country of origin				0.695
China	136 (46.6)	54 (48.6)	82 (45.3)	
Vietnam	65 (22.3)	26 (23.4)	39 (21.5)	
Philippines	22 (7.5)	6 (5.4)	16 (8.8)	
Japan	18 (6.2)	7 (6.3)	11 (6.1)	
Russia	14 (4.8)	8 (7.2)	6 (3.3)	
Other	37 (12.7)	10 (9)	27 (14.9)	
Age				0.021
≤ 29	115 (39.4)	55 (49.5)	60 (33.1)	
30-39	108 (37.0)	34 (30.6)	74 (40.9)	
≥ 40	69 (23.6)	24 (22.0)	47 (26.0)	
Marital status				0.281
Not married	79 (27.1)	34 (30.6)	45 (24.9)	
Married	213 (72.9)	77 (69.4)	136 (75.1)	
Education level				0.842
≤ HS graduate	181 (62.0)	68 (61.3)	113 (62.4)	
≥ College	111 (38.0)	43 (38.7)	68 (37.6)	
Religion^b^				0.227
No	116 (39.7)	49 (44.1)	67 (37.0)	
Yes	176 (60.3)	62 (55.9)	114 (63.0)	
Employment status				<0.001
Unemployed	157 (53.8)	74 (66.7)	83 (45.9)	
Employed	135 (46.2)	37 (33.3)	98 (54.1)	
Monthly income^c^				0.728
≤ 2 million KRW	215 (73.6)	83 (74.8)	132 (72.9)	
> 2 million KRW	77 (26.4)	28 (25.2)	49 (27.1)	
Korean language ability				0.148
Worse than average	182 (62.3)	75 (67.6)	107 (59.1)	
Better than average	110 (37.7)	36 (32.4)	74 (40.9)	
Length of stay in Korea				<0.001
≤ 4 years	149 (51.0)	75 (67.6)	74 (40.9)	
5-9 years	92 (31.5)	21 (18.9)	71 (39.2)	
≥ 10 years	51 (17.5)	15 (13.5)	36 (19.9)	
**Healthcare utilization and self health management characteristics**				
Have received healthcare service last year				0.011
No	52 (17.8)	28 (25.2)	24 (13.3)	
Yes	240 (82.2)	83 (74.8)	157 (86.7)	
Health insurance				0.571
Uninsured	89 (30.5)	36 (32.4)	53 (29.3)	
Insured	203 (69.5)	75 (67.6)	128 (70.7)	
Have regularly scheduled meals				0.824
No	187 (64.0)	68 (61.3)	119 (65.7)	
Yes	105 (36.0)	43 (38.7)	62 (34.3)	
Exercise regularly				0.315
No	236 (80.8)	93 (83.8)	143 (79)	
Yes	56 (19.2)	18 (16.2)	38 (21)	
Have interest in health maintenance				0.222
No	163 (55.8)	67 (60.4)	96 (53.0)	
Yes	129 (44.2)	44 (39.6)	85 (47.0)	
Perceived health status				0.974
Worse (than others)	195 (66.8)	74 (66.7)	121 (66.9)	
Better (than others)	97 (33.2)	37 (33.3)	60 (33.1)	

^a^Univariate logistic regression models were used to screen for possible predictors of CAM utilisation status.

^b^Participants were asked to select from pre-determined categories of religions or ‘none’ for no religion.

^*c*^Using exchange rates at the time of the study, two million Korean Won is equivalent to 1696 US dollars or 1370 Euros.

Table 2 presents modalities of CAM used by study participants. The most popular CAM modalities included multivitamin, ginseng, acupuncture, traditional Korean herbal medicines (han-yak), and hot spring bathing. In aggregate, CAM users employed a total of 768 CAM modalities. Most CAM users mentioned using multiple CAM modalities, averaging 4.25 *±* SD 2.08 modalities per CAM user. Nearly 70% of CAM users have visited and used one or more modalities associated with traditional Korean medicine (TKM). Popular methods of TKM used in this sampled group were acupuncture, moxibustion therapy, and herbal remedies. While assessing the most highly preferred CAM modalities in each ethnic group, we also found that ginseng was the preferred CAM modality among minorities coming from Pakistan, China, Mongolia, and Uzbekistan. Pakistani minorities in particular were the most frequent users of medicinal herbs and other natural remedies (i.e., ginseng, green juice, mushroom, and gingko). Of all manipulative treatment-based CAM modalities, acupuncture was the most frequently used among minorities from Mongolia.

**Table 2:** Modalities of CAM used by study participants

**Modalities of CAM**	**N**	**%**
Multivitamins	98	12.76%
Ginseng	92	11.98%
Acupuncture	91	11.85%
Traditional Korean herbal medicine	71	9.24%
Hotspring bath	67	8.72%
Massage therapy	61	7.94%
Brown rice	44	5.73%
Moxibustion	41	5.34%
Green vegetable juice	32	4.17%
Herbs	32	4.17%
Prayer/Relaxation	32	4.17%
Gingko for stimulation of peripheral circulation	18	2.34%
Yoga	16	2.08%
Cupping	15	1.95%
Mushrooms	14	1.82%
Sujichim, hand acupuncture	11	1.43%
Qigong	8	1.04%
Others	25	3.26%
Total no. of CAM modalities^a^	768	100.00%

*Note.*^a^Reflects total number of CAM modalities selected by respondents. Mean number of CAM modalities selected was 4.25 (*±* SD 2.08).

**Figure 1:**
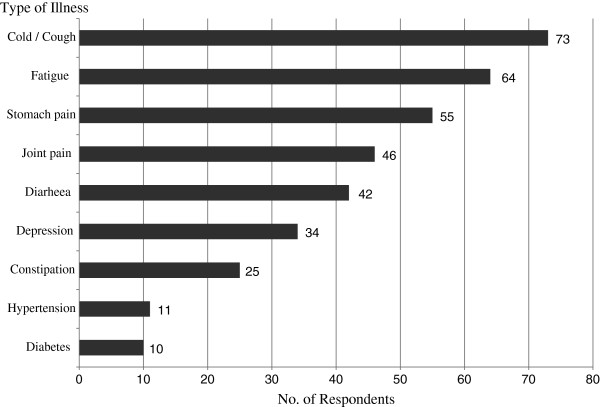
Acute vs. chronic: when or when not to consider cam use for self-health management.

While CAM is often used for self-management of existing medical conditions and health maintenance, it is important to know for which medical conditions people consider using CAM [21,22]. Thus, we provided study participants with nine common physical symptoms and medical conditions. Then, they were asked whether they would consider using CAM modalities for each of the listed conditions. In general, the majority of respondents considered CAM use for health management when dealing with acute diseases as opposed to chronic diseases such as diabetes and hypertension. As Figure 1 shows, cold, fatigue, stomach pain, and joint pain were the most frequently mentioned conditions for using CAM modalities among CAM users in the sample. About twenty percent of CAM users (N = 34) also reported that they would consider using CAM for mental health-related conditions such as depression. The most favoured CAM modalities in this particular group were traditional Korean medicinal herbs, acupuncture, ginseng, and multivitamins.

About half of CAM users mentioned that they use CAM because they believe in its effectiveness. Other reasons for using CAM were the following: the perception that conventional medicine has its limitations (23.8%), the perception that no harm will be done even if the CAM treatment is not effective (12.2%), and peer recommendation (8.3%). Most CAM users obtain information about CAM modalities through TKM clinics (29.3%) or a traditional pharmacy that specialises in medicinal herbs (45.3%). 55% of study participants felt that CAM was effective or that combining conventional medicine and CAM was more effective than conventional medicine alone. The treatment outcome that CAM users experienced included secondary treatment effects (18.9%), disease prevention and health promotion (30%), reduction in pain (16.1%), psychological stability (15%), and improved physical function (9.4%).

Table 3 and Table 4 show how study participants responded to questions regarding their CAM-related perceptions toward health beliefs and healthcare services. As expected, CAM users were more open-minded toward using CAM (18.8%) or integrating CAM with conventional medicine (45.3%) for health promotion and self-health management. For CAM users, CAM was also a trusted source for the treatment of disease (31%) and a safe alternative (38.6%). They also believed that it has made significant contributions toward advancing medical treatment (39.2%). About one third of CAM users agreed that doctors should have basic knowledge about traditional medicine outside of Korea. A similar percentage of CAM users (46.5%) and non-CAM users (42.3%) felt that it was important for doctors to know about a patient’s CAM usage.

**Table 3:** Selected survey questions on CAM-related beliefs and health services by CAM use status

**Variables**		**Total number of respondents N (%)**	**Non-CAM user n (%)**	**CAM user n (%)**	***p-value***
		**292 (100.0)**	**111 (100.0)**	**181 (100.0)**	
Of the following choices, which do you think is most effective for health promotion and self-management purposes?	Conventional medicine	67 (22.9)	33 (29.7)	34 (18.8)	<0.001
	CAM	43 (14.7)	9 (8.1)	34 (18.8)	
	CAM + Conventional medicine	117 (40.1)	35 (31.5)	82 (45.3)	
	Don’t know	65 (22.3)	34 (30.6)	31 (17.1)	
Do you trust CAM for the treatment of diseases?	Strongly distrust	12 (4.1)	7 (6.3)	5 (2.8)	<0.001
	Distrust	36 (12.3)	21 (18.9)	15 (8.3)	
	Neutral	175 (59.9)	70 (63.1)	105 (58)	
	Trust	55 (18.8)	12 (10.8)	43 (23.8)	
	Strongly trust	14 (4.8)	1 (0.9)	13 (7.2)	
Do you agree that CAM has made contributions toward advancing medical treatment?	Strongly disagree	17 (5.8)	9 (8.1)	8 (4.4)	<0.001
	Disagree	36 (12.3)	23 (20.7)	13 (7.2)	
	Neutral	157 (53.8)	68 (61.3)	89 (49.2)	
	Agree	72 (24.7)	10 (9.0)	62 (34.3)	
	Strongly agree	10 (3.4)	1 (0.9)	9 (4.9)	
Do you feel that CAM treatments are safe?	Strongly disagree	14 (4.8)	9 (8.1)	5 (2.8)	<0.001
	Disagree	36 (12.3)	14 (13.0)	21 (11.6)	
	Neutral	156 (53.4)	71 (64.0)	85 (47.0)	
	Agree	74 (25.3)	16 (14.0)	58 (32.0)	
	Strongly agree	12 (4.1)	1 (0.9)	12 (6.6)	
When you receive medical care, do you agree that doctors should be informed about your use of CAM?	Strongly disagree	17 (5.8)	10 (9.0)	7 (3.9)	0.371
	Disagree	44 (15.1)	14 (12.6)	30 (16.6)	
	Neutral	100 (34.2)	40 (36.0)	60 (33.1)	
	Agree	97 (33.2)	35 (31.5)	62 (34.3)	
	Strongly agree	34 (11.6)	12 (10.8)	22 (12.2)	
When you receive medical care, do you agree that doctors need to have basic understanding of traditional medicine from your home country?	Strongly disagree	18 (6.2)	7 (6.3)	5 (2.8)	<0.001
	Disagree	32 (11)	21 (18.9)	15 (8.3)	
	Neutral	89 (30.5)	70 (63.1)	105 (58)	
	Agree	119 (40.8)	12 (10.8)	43 (23.8)	
	Strongly agree	34 (11.6)	1 (0.9)	13 (7.2)	

**Table 4:** **Selected survey questions on CAM-related beliefs and health services by CAM publicly accepted in the country of origin**^**a**^

**Variables**		**Total number of respondents N (%)**	**No n (%)**	**Yes n (%)**	***p-value***
		**292 (100.0)**	**81 (100.0)**	**211 (100.0)**	
Of the following choices, which do you think is most effective for health promotion and self-management purposes?	Conventional medicine	67 (22.9)	19 (23.5)	48 (22.7)	0.079
	CAM	43 (14.7)	5 (6.2)	38 (18.0)	
	CAM + Conventional medicine	117 (40.1)	36 (44.4)	81 (38.4)	
	Don’t know	65 (22.3)	21 (25.9)	44 (20.9)	
Do you trust CAM for the treatment of diseases?	Strongly distrust	12 (4.1)	4 (4.9)	8 (3.8)	0.198
	Distrust	36 (12.3)	14 (17.3)	22 (10.4)	
	Neutral	175 (59.9)	45 (55.6)	130 (61.6)	
	Trust	55 (18.8)	17 (21)	38 (18.0)	
	Strongly trust	14 (4.8)	1 (1.2)	13 (6.2)	
Do you agree that CAM has made contributions toward advancing medical treatment?	Strongly disagree	17 (5.8)	10 (12.3)	7 (3.3)	0.026
	Disagree	36 (12.3)	12 (14.8)	24 (11.4)	
	Neutral	157 (53.8)	39 (48.1)	118 (55.9)	
	Agree	72 (24.7)	19 (23.5)	53 (25.1)	
	Strongly agree	10 (3.4)	1 (1.2)	9 (4.3)	
Do you feel that CAM treatments are safe?	Strongly disagree	14 (4.8)	7 (8.6)	7 (3.3)	0.275
	Disagree	36 (12.3)	12 (14.8)	24 (11.4)	
	Neutral	156 (53.4)	40 (49.4)	116 (55.0)	
	Agree	74 (25.3)	20 (24.7)	54 (25.6)	
	Strongly agree	12 (4.1)	2 (2.5)	10 (4.7)	
When you receive medical care, do you agree that doctors should be informed about your use of CAM?	Strongly disagree	17 (5.8)	9 (11.1)	8 (3.8)	<0.001
	Disagree	44 (15.1)	17 (21)	27 (12.8)	
	Neutral	100 (34.2)	34 (42)	66 (31.3)	
	Agree	97 (33.2)	19 (23.5)	78 (37.0)	
	Strongly agree	34 (11.6)	2 (2.5)	32 (15.2)	
When you receive medical care, do you agree that doctors need to have basic understanding of traditional medicine from your home country?	Strongly disagree	18 (6.2)	5 (6.2)	13 (6.2)	<0.001
	Disagree	32 (11)	19 (23.5)	13 (6.2)	
	Neutral	89 (30.5)	28 (34.6)	61 (28.9)	
	Agree	119 (40.8)	23 (28.4)	96 (45.5)	
	Strongly agree	34 (11.6)	6 (7.4)	28 (13.3)	

*Note.*^a^Respondents from countries that receive public support for traditional medicine (i.e., China, Japan, Vietnam, Sri Lanka, and Mongolia) were compared with the rest.

In Table 4, we compared selected survey responses to CAM-related perception of those whose home country publicly supports or does not support CAM. More than 52% of people from countries that support CAM discussed their CAM use with physicians. They also wanted Korean physicians to have a basic understanding of traditional medicine use in their home country (58.8%), believed that CAM had made significant contribution toward advancement in medicine (29.4%), and believed that CAM is a safe alternative to conventional medicine (30.3%).

## Discussion

The prevalence of CAM use was considerably high among ethnic minorities in Korea. In our study, we found that 62% of ethnic minorities were CAM users. When compared to Korean adult CAM use rate of 75% and the rates of minority CAM use reported from other Asian countries (i.e., Malaysia and Singapore), the prevalence rate was slightly lower; however, it was much higher than those reported in U.S. studies [4,6,8-11,23]. We also found that the overall rates of CAM use and preferred CAM modalities varied based on ethnicity. When ethnicity was taken into account, CAM use rates fluctuated from 42-100% within our study. High CAM use rates were found in respondents from the following countries: Pakistan, Mongolia, Philippines, and Cambodia. While past studies have found high proportion of active CAM users in those countries [10,11,24,25], our study demonstrated that they continue to use CAM or practice traditional medicine in the receiving country.

Similar to past studies, potential predictors of CAM use found in our sample include female gender, younger age, and having regular healthcare [3,4,6,8]. Because the Korean government allows all documented aliens to register for national health insurance, it is not surprising that most of our participants had regular source of care [26]. Recent immigration was also a potential predictor of CAM use in our sample. In contrast to the U.S. study that did not find a statistically significant relationship, CAM use and recent immigration had an inverse relationship in our sample [9]. Only 70% of study participants had health insurance at the time of the interview, and most were recent immigrants. Thus, this study suggested that ethnic minorities may rely heavily on CAM use because they may be facing barriers in access to care due to difference in culture and the healthcare system [19].

Close to twenty percent of CAM users in our sample mentioned that they would use CAM modalities for mental health problems. As other U.S. studies have previously found, many ethnic minorities were using multiple CAM therapies to treat mental health problems such as anxiety disorder and depression [27-30]. In conventional psychiatric practice, anti-psychotic polypharmacy has raised safety concerns regarding drug interactions [31]. While clinical testing and approval of CAM therapies is still lacking, clinicians might encounter unwanted adverse effects and complications in CAM users who are simultaneously being treated with conventional psychiatric treatment. Furthermore, there have been reports of toxic contaminants and inadequate product information labelling in herbal medicine from China, the world’s largest distributor of herbal products [32,33]. Ironically, Korea is one of the top importers of herbal medicine from China. Knowing that immigrants might be at a higher risk of mental health problems due to acculturation stress and other social factors, clinicians need to be aware of CAM use among minorities. However, language and cultural barriers between physician and ethnic minorities could hinder communication and produce unwanted medical complications. Thus such communication barriers will need to be resolved to promote ethnic minorities’ psychological wellbeing and reduce preventable medical accidents [34].

Our study has number of limitations. First, our findings do not represent the CAM use status of all ethnic minorities in Korea. In the absence of a probability sample, we collected a small convenience sample of ethnic minorities from a suburban location. Due to the high cost of living in urban centres, ethnic minority communities in Korea are often formed in sub-urban cities near the subway system. Thus, we may have oversampled minorities in the lower income bracket, and this potential bias was reflected in our data (i.e., 73.6% of participants made 2 million KRW or less). Secondly, the majority of study participants were middle-aged women and recent immigrants who had lived in Korea for less than 10 years. Nevertheless, our pilot study has generated many new insights about CAM use among ethnic minorities in Korea. Future studies should continue to investigate this group using a larger, more socioeconomically and ethnically diverse sample.

## Conclusion

There is considerably high prevalence of CAM use among ethnic minorities in Korea. They perceive combined use of CAM and conventional medicine as beneficial for their overall health. Many CAM users reported that they want doctors to know about their CAM use and have a basic understanding of traditional medicine in their home country. Physicians need to be aware of the fact that many ethnic minorities use the modalities of CAM. However, language and cultural barriers between physician and them could hinder communication and produce unwanted medical complications. Overcoming the barriers could help promote their psychological wellbeing and prevent medical complications. High prevalence of CAM use among ethnic minorities in our study warrants further studies using larger sample population.

## Competing interests

The authors declare that they have no competing interests.

## Authors’ contributions

DW was responsible for conception and design, analysis and interpretation of the data, and drafted the manuscript. HJ provided feedback in each phase of the study and helped draft the manuscript and critically revised the manuscript. All authors read and approved the final manuscript. EY and WK participated in the study design for collection of supplement data and were involved in participant recruitment.

## Pre-publication history

The pre-publication history for this paper can be accessed here:

http://www.biomedcentral.com/1472-6882/14/103/prepub
